# Study of Segregation in Non-Dilute Solutions of Linear Diblock Copolymers and Symmetric Miktoarm (or Janus Star) Polymers Using Monte Carlo Simulations with the Bond Fluctuation Model

**DOI:** 10.3390/polym13142377

**Published:** 2021-07-20

**Authors:** Juan J. Freire

**Affiliations:** Departamento de Ciencias y Técnicas Fisicoquímicas, Facultad de Ciencias, Avenida de Esparta s/n, 28232 Las Rozas de Madrid, Spain; jfreire@invi.uned.es

**Keywords:** diblock copolymers, miktoarm polymers, Janus star polymers, Monte Carlo simulations, bond fluctuation model

## Abstract

The bond fluctuation model was employed to characterize the approach to the mesophase separation transition of pure linear AB copolymers and symmetric miktoarms, also called Janus, star polymers, A_*f*/2_B_*f*/2_, where *f* = 6 or 12 is the total number of arms, in a common good solvent. We consider a concentration sufficiently high to mimic the melting behavior and also a lower concentration. The segregation between A and B units is represented by a repulsive interaction parameter, ε. Different total numbers of units are also considered. Results for different properties, such as the molecular size, the asphericity and orientational correlation of blocks, or arms, of different compositions are obtained as a function of the segregation parameter. We also calculate scattering structure factors. The initial effect of segregation on the scattering with opposite contrast factors between the A and B blocks can be explained with a common description based on the random phase approximation for both the linear copolymers and the *f* = 6 miktoarms, once the numerical form factors of the different molecules in their particular systems are considered. However, the results for *f* = 12 clearly deviate from this description probably due to some degree of ordering in the position of highly armed molecules.

## 1. Introduction

A*_n_*B*_m_* miktoarm polymers [1,2] are molecules composed of *n*+*m* arms of homopolymers with different repeat units, A and B, joined to a common core or central units. They show peculiar properties because of the segregating heterointeraction between arms of different types. Therefore, their behavior is different from that of the (AB)*_n_* diblock arm star polymers where each one of the n arms is constituted by a diblock AB polymer. The symmetric A*_n_*B*_n_* molecules are also known as Janus stars [3,4], since the A and B units tend to symmetrically align in different directions, in arrangements similar to those observed in other Janus nanostructures [5].

Polymers composed of different blocks have a transition from the disordered state to form mesophases due to segregation between the block. This microphase separation transition (MST) is the subject of theoretical [6,7,8] and numerical simulation [9,10,11] studies. Leibler [6] applied a mean-field theory for ideal diblock copolymers some years ago, characterizing different types of mesophases whose formation depends on the copolymer composition and thermodynamic conditions. In the case of symmetric diblocks, the theory predicts a single transition to lamellar structure located at (*χN*)_MST_ ≅ 10.5, where *χ* and *N* are the thermodynamic parameter and number of polymer units as defined in the Flory–Huggins (FH) theory. A similar study was subsequently accomplished by Olvera de la Cruz and Sanchez [8] for different types of star copolymers. In the case of ideal symmetric A*_n_*B*_n_*, or Janus, stars with arms of *N*/2*n* A or B units, a transition to a lamellar mesophase is similarly found at [*χ*(*N*/*n*)]_MST_ ≅ 10.5.

The mean-field results can be simply described through the prediction of the random phase approximation (RPA) [12,13,14] for the symmetric diblock copolymer scattering structure factor or normalized scattering intensity, *I_AB_*(*q*),

(1) $$I_{AB}^{}\left(q\right)=1/N\Phi P_{AB}^{}\left(q\right)-\chi /2$$

where *P_AB_*(*q*) is the ideal copolymer form factor of a single molecule (in the absence of segregation effects and assuming Gaussian statistics) and *Φ* is its volume fraction introduced as a correction when the molecule is immersed in a common, optically neutral solvent, for A units and B units. For the case of symmetric molecules in the melt, the copolymer form factor is obtained assuming the opposite unit contrast factor of 1 and −1 for the A and B units. The same choice can be applied to copolymers immersed in an optically neutral solvent. *P_AB_*(*q*) is calculated from the vectors that connect the positions of each pair of units *i* and *j* (A-A, B-B or A-B) [15]:

(2) $$P_{AB}^{}\left(q\right)=(1/N^{2})\left[\sum_{iA}^{N/2}\sum_{jA}^{N/2}e^{iq.(R_{iA}-R_{jA})}+\sum_{iB}^{N/2}\sum_{jB}^{N/2}e^{iq.(R_{iB}-R_{jB})}-\sum_{iA,iB}^{N/2}\sum_{jB,jA}^{N/2}e^{iq.(R_{i}-R_{j})}\right]$$

The mesophase transition can be characterized by a divergence of *I_AB_*(*q*) at a given value of the scattering variable, *q_max_*. Therefore, the corresponding value of the Flory–Huggins parameter is given by:

(3) $$N\chi_{TMS}\Phi =2/P_{AB}^{}\left(q_{\max}\right)$$

For a symmetric diblock copolymer it is shown that:

(4) $$P_{AB}\left(q\right)=P_{N/2}\left(q\right)-P_{N}\left(q\right)$$

Finally, with the consideration of Gaussian statistics, one obtains *P_AB_*(*q_max_*) ≅ 0.190. When this value is introduced in Equation (1) it leads to the Leibler result for (*χN*)_MST_ in the melt case, *Φ* = 1.

Similarly, we verified that the form factor of an AnBn miktoarm can be obtained as:

(5) $$P_{AB}\left(q\right)=\left(1/n\right)\left[P_{N/2}\left(q\right)-P_{N}\left(q\right)\right]$$

This also leads to the melt value for [*χ*(*N*/*n*)]_MST_ obtained by Olvera de la Cruz and Sanchez. We use this approach to introduce corrections in the case of non-ideal molecules for which a numerical evaluation of the form factor can be easily accomplished.

More recently, a renormalized one-loop (ROL) theory [14,16,17] was able to introduce corrections to the peak intensity and location of the structure-function of diblock copolymers, describing how the variation of 1/*I_AB_*(*q*) with *χN* shows a deviation upwards from the initial linear behavior when the systems approach the MST. This theory was shown to give a good description of simulation data showing the approach to the MST transition of symmetric linear diblock copolymers in the disordered melt state.

In the present work, we present Monte Carlo simulations for linear diblock copolymers, *f* = 2, and symmetric miktoarms composed of *f* = 6 and *f* = 12 arms, or *n* = 3 and *n* = 6, [18,19] in solution, with different values of the interaction parameter, number of units and concentration in a common, implicit, good solvent. We use the bond fluctuation model (BFM) [20,21] that permits the study of systems composed of a relatively large number of polymer molecules. Deviations from Gaussian statistics are expected in the case of the star polymers due to the presence of a central core. The highest concentration considered in this work represents a system close to the melting behavior. The aim of this study is to verify whether the different topologies, number of polymer units and concentrations may exhibit a common initial approach to the MST and to establish the way to use the RPA description for this purpose

## 2. Numerical Methods

We introduce *n_t_* molecules, each of *N* units, in a cubic lattice of length *L*. The copolymers are composed of two blocks of *N*/2 units. Each symmetrical star molecule contains (*N*−1)/2 A units, (*N*−1)/2 B units and a central unit. The A and B units are distributed into *f* arms, constituting a symmetrical star molecule. *b* is the length unit and corresponds to the distance between adjacent lattice sites. According to the BFM specifications [20], each bead occupies a site and it also blocks its closest 26 sites. This fulfills the self-avoiding walk (SAW) condition. Bond lengths are all possible connections between sites in the range between 2 and 10^1/2^, but the value 8^1/2^ is avoided because bonds of the type (±2 ±2 0) may cross each other during the simulation. Furthermore, we consider a distance-dependent energy term between A and B beads of non-bonded units [10,21,22] whose sites are at a distance smaller than 10^1/2^. This energy is multiplied by a factor, *ε*, in units relative to the Boltzmann factor *k_B_T*. Positive values of *ε* gauge a net repulsion between A and B units. Therefore, *ε* is proportional to *χ*. Box lengths are fixed in the range *L* = 92–112, which is high enough to avoid a significant number of interactions between the replicas of any unit of a given molecule that can be generated by the application of periodic boundary conditions. *n_t_* is fixed to comply with the fixed polymer volume fraction. According to the blocked site specifications, a molecule unit effectively occupies 8 sites, *Φ* = 8*nN*/*L*^3^.

The initial configurations are constructed by building a regular arrangement of molecules, leaving sufficient unoccupied beads between them so as to allow for an efficient and complete equilibration. The details about this procedure for linear chains and star polymers are specified elsewhere [23]. From these initial configurations, the simulations run over a given number of Monte Carlo simple bead jumps. Each bead jump consists of the displacement of a single unit to one of its closest neighboring sites. The jump is accepted if it complies with the bond distance specifications and the SAW condition with respect to non-bonded units, also taking into account the variation in energy according to the Metropolis criterion. Consistently with this criterion, the previous configuration is again considered if a jump is rejected. A step corresponds to *n_t_N* jump attempts, after which each unit has a single statistical chance to move. We introduce a certain number of equilibration steps (2 × 10^6^) and properties are collected every 4000 steps. The simulations are extended to obtain averages on 1000 to 7000 property values, depending on the particular system.

We consider two values of the total number of units, *N* = 72 or 144 for the diblock copolymers and *N* = 73 or 145 for the *f* = 6 or 12-star molecules. We also consider two different volume fractions *Φ* ≅ 0.275 or *Φ* ≅ 0.135 for the different types of molecules. The former value may correspond to systems close and behaving similarly to melts as will be discussed below. Therefore, we study the properties of 12 different systems, each with varying values of the parameter *ε*.

We calculate properties that indicate the progressive segregation between A and B units. The acceptance ratio of jumps, *a_r_*, gives useful information about the system behavior. In addition, we obtain the averaged scalar product of the vectors defined by the molecule center and the two ends of the diblock copolymers or between the central unit and the end unit in arms of different A and B types for the miktoarms.

(6) $$C_{AB}=\left\langle \left(v_{ce}^{A}.v_{ce}^{B}\right)/v_{ce}^{2}\right\rangle {\;\mathrm{where}\;v}_{ce}^{2}={\left\langle {\left(v_{ce}^{A}\right)}^{2}{\left(v_{ce}^{B}\right)}^{2}\right\rangle }^{1/2}$$

We also examine the asphericity [24,25], i.e., the deviation from a spherical shape, defined as:

(7) $$A=\frac{\left\langle \sum_{i>j}^{3}{\left(\lambda_{i}-\lambda_{j}\right)}^{2}\right\rangle }{\left\langle 2{\left(\sum_{i=1}^{3}\lambda_{i}\right)}^{2}\right\rangle }$$

where λ*_i_* is the *i*^th^ eigenvalue of the radius of gyration tensor. Moreover, we obtain the molecule size and extension, represented by the averaged mean quadratic radius of gyration, $R_{g}^{2}$, and the averaged mean quadratic distance between end units, $\langle R_{ee}^{2}\rangle$.

Furthermore, we estimate the average number of repulsions between A and B units as <*E_rep_*>/*ε*, where *E_rep_* is the repulsion energy obtained for a given configuration. The results corresponding to *ε* = 0 are obtained as limits from data obtained with very small repulsion.

As stated in the Introduction, a quantification of the approach to the MST that can be directly compared with the RPA is provided by the structure factor, a property that can also be related to scattering experiments. As with our computation of the form factor, we can obtain this property assuming opposite, 1 and −1, optical contrast factors for units A and B, but now using vectors connecting the pairs of sites, *i_A_* or *i_B_*, occupied by all the units in the lattice, taking into account the total number of sites actually blocked [22]:

(8) $$I_{AB}(q)=(8/L^{2})\left[\sum_{i_{A}}^{L}\sum_{j_{A}}^{L}e^{iq.\left(R_{iA}-R_{jA}\right)}+\sum_{i_{B}}^{L}\sum_{j_{B}}^{L}e^{iq.\left(R_{iB}-R_{jB}\right)}-\sum_{i_{A},i_{B}}^{L}\sum_{j_{B},j_{A}}^{L}e^{iq.\left(R_{iA}-R_{iB}\right)}\right]$$

We assume that sites that are not occupied or blocked by the molecule units are occupied by the solvent and they do not contribute to the total scattering. As explained in the case of the form factor, this is obviously true if the concentration of the systems is high enough to be representative of a melt from the optical point of view. Otherwise, it can only be related to a scattering experiment if the refractive index of the solvent is chosen to give a null scattering contribution.

We also checked the global distribution of the molecules along the systems not directly related to the arrangement of A and B units. To this end, we computed the alternative structure function:

(9) $$I_{ps}(q)=(8/L^{2})\sum_{i}^{L}\sum_{\begin{array}{l} j_{} \end{array}}^{L}f_{i}e^{iq.R_{ij}}$$

where *f_i_* = (1 − *Φ*/8) if the site contains a unit or *f_i_* = − *Φ*/8 otherwise [22]. This function gauges any heterogeneity in the distribution of copolymer chains or miktoarm molecules in the box, considering the same optical contrasts for the A and B units with respect to the solvent. The appearance of peaks in *I_ps_*(*q*) can be related to some degree of ordering or can reflect segregation between the molecules and solvent (or empty sites). The latter effect can only be present in models where attractive AA and BB interactions are introduced to describe net AB repulsions but it is not possible for the present model with purely repulsive AB interactions.

## 3. Results and Discussion

Table 1 contains the numerical results obtained for properties for diblock copolymers, *N* = 144, and miktoarms, *N* = 145, and the higher volume fraction, *Φ* ≅ 0.275, including segregation, together with data corresponding to *ε* = 0 for other systems. Comparing values obtained with the different systems without segregation we observe that, as expected, the acceptance ratio is greater for lower concentration and number of units and it has a weak dependence on *f*. *C_AB_* shows a slightly negative value even without segregation because of the mutual exclusion of different blocks. Its absolute value decreases with *f* as the number of contributions to the average from different blocks increases. The asphericity has a remarkable decrease with *f* as molecules with a high number of units adopt a shape close to spherical and also a decrease for the systems with a smaller number of units and density. Ratios $\langle R_{ee}^{2}\rangle /R_{g}^{2}$ are close to the expected values of six for linear chains and two for star molecules. Additionally, averaged quadratic sizes are expected to be proportional to the number of units, but the results for the lower densities clearly show a further increase with *N* due to excluded volume effects associated with the presence of a good solvent, which is more significant for the linear chains. This effect also causes an increase in the size with density for the lower density systems that can be observed in all the cases. Finally, the number of repulsions also obeys the expected behavior, increasing with *f* and decreasing with density.

The different properties also vary with increasing repulsion between A and B units, or increasing values of *ε*. Thus, the acceptance ratio decreases as the A and B units adopt more stable distributions. The decrease is more dramatic for the miktoarms, which initially suffer stronger hindrances due to the arm constraints than for the diblock copolymers. The averaged scalar product between center and end units, *C_AB_*, is always negative and its absolute value increases with segregation as units of the same type tend to align. Additionally, it is significantly greater for the diblock copolymers in absolute values because the segments are less constrained. However, the effect is similar in percentage for the different molecules. Asphericity increases with the segregation between A and B units since similar units align. The relative effect is more pronounced for the miktoarms, especially in the case of the *f* = 12 molecules, whose shape in absence of segregation is closer to spherical for a similar total number of A and B units. The size of molecules, characterized by $R_{g}^{2}$ and $\langle R_{ee}^{2}\rangle$, relates with asymmetry and also increases with segregation, especially in the case of the more flexible diblock copolymers. A substantial decrease in the number of net repulsions can be observed when segregation increases as the molecules adopt more ordered configurations. In summary, more pronounced variations are observed for the highest values of *ε* for all these properties, though these changes are not sharp enough in any range of values of *ε* to give a precise indication of the MST location.

Our values of *I_AB_*(*q*) for each given system are analyzed to determine its maximum value and its location. Both *I_AB_*(*q_max_*) and *q_max_* are expected a systematic variation with *ε*. The maximum scattering should show a dramatic increase when the systems approach the MST, as predicted by the RPA. Moreover, *q_max_* suffers a small decrease as segregation increases. The ROL theory predicts a maximum decrease of about 20% for segregated simulation data of diblock copolymers [16], that it is somehow reproduced in the present systems. We also observe a similar decrease showing an earlier onset for the highly armed molecules. This can be related to the increase in intermolecular interactions.

In the absence of interactions between units, it is expected that the structure factor can be simply obtained by adding the individual molecular contributions represented by the form factor according to the expression:

(10) $$I_{AB}(q,\varepsilon =0)=N\Phi P_{AB}(q)$$

The values for the form factor obtained from Equation (10) with our simulations for star polymers do not agree with the results obtained for the ideal star molecules, implicit in the Olvera de la Cruz and Sanchez predictions, due to the finite size of the chains. The BFM is able to describe the restricted disposition of the units belonging to different arms in the bulky core near the star center. More external A and B units cannot easily access this densely packed core which induces a greater degree of exclusion between different arms. In Table 2, the results *P_AB_*(*q*) obtained from *I_AB_*(*q_max_*) and Equation (10) for *Φ* ≅ 0.275 are compared with the average of the form factor values that can be directly calculated from individual molecules, <*P_AB_*(*q_max_*)>. Deviations from ideality are small for both sets results in the case of the diblock copolymers, but they are more significant for the *f* = 6-star molecules. It can also be observed that the form factors from Equation (10) are greater than the <*P_AB_*(*q_max_*)> values for this particular case. This can be explained because A and B units belonging to other molecules are also excluded from the bulky core. Moreover, correlations in the disposition of units corresponding to separate molecules with a central core are possible as they were observed in the case of non-dilute solutions of dendrimers [26]. 

**Figure 1:**
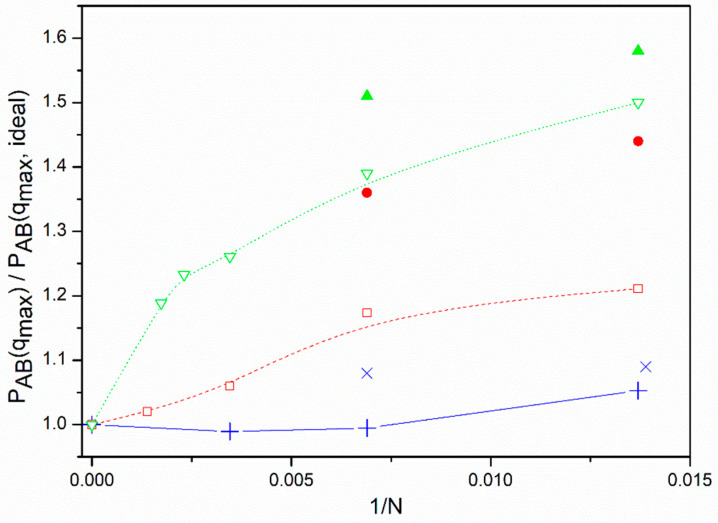
Ratio between the simulation form factors obtained from simulations with the higher density systems, *Φ* ≅ 0.275, and the predictions for ideal chains. Results for single chains obtained with a non-lattice model: solid blue line and blue + crosses, *f =* 2; dashed red line and open red squares, *f* = 6; dotted green line and open green triangles down, *f* = 12. Results from simulation data obtained with the BFM and Equation (10), *ε* = 0: blue x crosses, *f* = 2; filled red circles: *f* = 6; filled green triangles up, *f =* 12.

Using a different model previously employed for the study of single linear and star polymers [27,28] (off-lattice with Gaussian distribution of distances between neighboring units and both attractive and repulsive long-range interactions, set to reproduce the theta conditions), we also study the approach of *P_AB_*(*q_max_*) to the pseudo-ideal results of individual molecules with a greater number of units. The results relative to the ideal limit values are compared with the BFM data corresponding to our highest volume fraction in Figure 1. It is observed that the star molecules tend to reach the ideal values only when the number of units is remarkably high, while this approach is much faster in the case of linear chains. It can also be observed that the approach of the star molecules to their limits is slower for the non-diluted systems. Thus, the results are significantly higher than the ideal limit for some of the systems with the studied number of units. Since the number of monomers represented by a unit in a given model depends on the polymer characteristics (in particular, of its Kuhn length as the first estimation of its rigidity), and the presence of other chains reinforces the exclusion of units from the core in non-dilute systems, it is possible that real star polymers of relatively high molecular weight in non-dilute solutions or melts cannot reach the ideal value for *P_AB_*(*q*). Consequently, real miktoarm molecules may show deviations from the RPA results for ideal molecules simply because of finite size effects induced in their form factors by the presence of a bulky core and its influence on intermolecular interactions.

**Figure 2:**
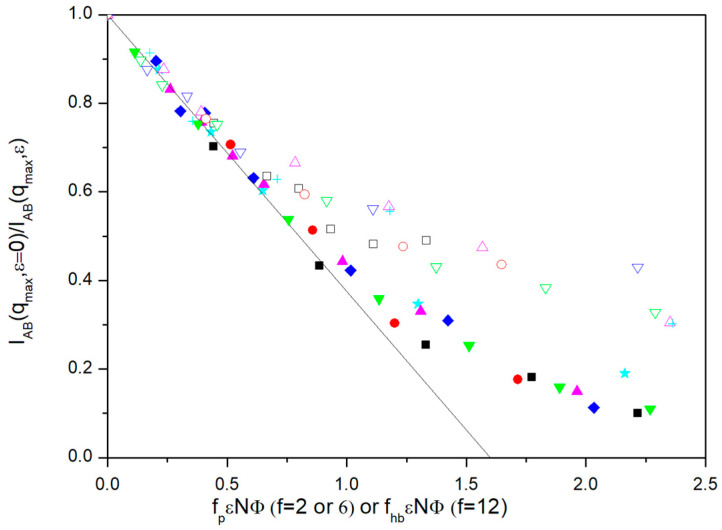
Ratio between scattering functions obtained with opposite optical contrast factors for A and B units without segregation and with different values of *ε* vs. *f_P_εNΦ* for *f* = 2 and *f* = 6, or *f_hb_εNΦ* for *f* = 12, see text. Blue rhombuses, *N =* 144, *f =* 2; red circles: *N = *145, *f = *6; magenta triangles up: *N =* 145, *f =* 12; cyan stars: *N =* 72, *f =* 2; black squares, *N =* 73, *f = 6*, green triangles down: *N =* 73, *f =* 12. Filled symbols correspond to *Φ* ≅ 0.275 and open symbols correspond to *Φ* ≅ 0.135. Solid black line: best fit of the points in the linear region of low *ε* values.

The values of *I_AB_*(q*_max_*) and their variation with the parameter describing segregation, *ε*, deserve particular attention as they can be directly compared with the accuracy of the RPA to give a quantitative description of the approach to the MST. With this aim, we normalized these results with respect to the data obtained for *ε* = 0. In Figure 2, we show the variation of *I_AB_*(*q_max_*,*ε* = 0)/*I*_AB_(*q_max_*,*ε*) with *f_P_εNΦ* for the different molecules, the number of units and volume fractions. *f_p_* is a numerical constant for each system that takes into account the deviation of the form factor at *q*_max_ for the systems that do not include segregation, evaluated from Equation (10), from the ideal value corresponding to diblock copolymer molecules (that is taken as a reference), *P_AB_*(*q_max_*) ≅ 0.190,

(11) $$f_{p}=I_{AB}(q_{\max},\varepsilon =0)/0.190N\Phi$$

*f_p_* incorporates both the purely topological differences between the ideal diblock copolymers and miktoarms expected from the application from Equations (4) and (5) even in the case of ideal molecules and also the deviations from Gaussian statistics due to the presence of the bulky star cores and related intermolecular effects.

In the simplest RPA description, Equations (1) and (3) predict a linear variation of *I_AB_*_(_*q_max_*,*ε* = 0)/*I_AB_*(*q_max_*,*ε*) reaching the value 0 at the MST. The ROL theory provides a more detailed description [14,16,17], reflecting an upward deviation of the data with respect to the RPA prediction as the systems approach MST. This feature has previously been confirmed in previous Monte Carlo simulations for diblock copolymers in the melt state. Our data qualitatively obey this behavior but, given the diverse nature and complexity of the systems investigated, we are not attempting here to make a quantitative comparison of our simulation data with this theory.

It can be observed that all the diblock copolymer and the *f* = 6 miktoarm data show the expected linear behavior in the range of small values of *ε*. Taking into account statistical fluctuations, the data do not show a systematic variation with the number of units or fraction volume. It should be noted that the ROL theory predicts a small but noticeable dependence of the 1/*I*_AB_(*q_max_*) vs. *χN* curves with the number of units in the whole interval of *χN* values. The actual extent of this dependence depends on the particular type of model. Some accurate simulations for diblock copolymers melt also show a model dependence on *N*, though it is actually smaller than the theoretical prediction except for some particular cases [16].

Results for the two different volume fractions employed in our simulation are roughly grouped for small *ε* values. However, the results for lower values of *Φ* exhibit an earlier upwards deviation, consequently showing a greater difficulty to achieve the MST, as it can be intuitively expected. Moreover, the results seem to be practically independent of topology, when considering the linear chains and the *f* = 6 miktoarms, once the simulation results for *P_AB_*(*q_max_*), describing the star core finite size and its influence on intermolecular effects, are introduced in the factor *f_p_.*

The common linear initial behavior shown by the diblock and *f* = 6 miktoarm molecules can be extrapolated to the point where *I_AB_*(*q_max_*,*ε* = 0)/*I_AB_*(*q_max_*,*ε*) = 0. This way, our simulations predict a common *f_p_εNΦ* value for the theoretical prediction of the MST according the RPA, (*f_p_εNΦ*)_MST_ ≅ 1.60. This value can be discussed taking into account the particular features of the BFM. Previous simulations for polymer-solvent systems with this model were able to establish that the theta state (parameter *χ* = 1/2 in the FH theory) corresponds to a value *ε_PS_* ≅ 0.2 for the attractive interactions between units in a homopolymer chain [29]. The definitions of interactions in the FH theory are consistent with considering *ε = ε_PS_*/2, for attractive or repulsive interactions. Therefore, we can assume *χ* ≅ 5*ε* and, therefore, (*χNΦ*)_MST_ ≅ 8.0. It was previously discussed that the value *Φ* = 1 in the BFM does not correspond to the melt state since a fully occupied lattice does not allow for any motion of the units. Actually, the alternative value *Φ* = 0.5 was proposed as a more accurate representation of the melting behavior [30]. Taking into account the RPA theoretical value, *f_p_*(*χN*)_MST_ ≅ 10.5, the present simulation data are consistent with a smaller effective value for the BFM melt state, *Φ*_melt_ ≅ 0.36.

**Figure 3:**
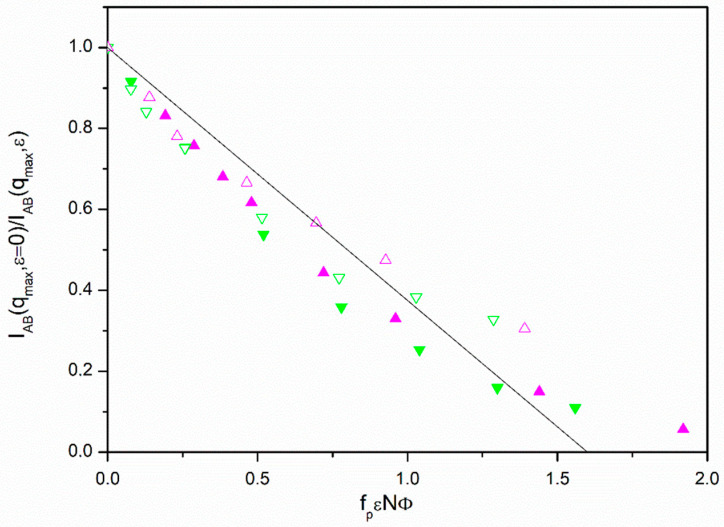
Ratio between the scattering functions obtained with opposite optical contrast factors for A and B units without segregation and with different values of scattering functions obtained with opposite optical factors for A and B units without segregation and with different values of *ε* vs. *f_P_εNΦ* for *f* = 12. Magenta triangles up: *N =* 145; green triangles down: *N = *73. Filled symbols correspond to *Φ* ≅ 0.275 and open symbols correspond to *Φ* ≅ 0.135. Solid black line: best fit of the points of Figure 2 in the linear region of low *ε* values.

The results for *f* = 12, however, cannot be treated in a similar way. In Figure 3, we show that all these points clearly deviate downwards from the RPA prediction when we employ the same representations used in other cases. In Table 2 we can observe that the form factor results obtained from Equation (10) and the averages <*P_AB_*(*q_max_*)> roughly agree for *f* = 12 molecules. However, in the absence of additional effects, we would expect greater core differences between the form factors obtained from Equation (10) and the averages <*P_AB_*(*q_max_*)> for the *f* = 12 molecules than those calculated from the *f* = 6 form factor results. Therefore, the intermolecular effects on the exclusion of A or B units from the core shown by th*e f* = 6 chains were somehow eliminated even though the cores are assumed to be bulkier for stars with more arms. Consistently, it can be observed in Figure 1 that the discrepancy between the form factor results for non-diluted systems and those found for single chains is greater for *f* = 6 than for the *f* = 12 cases.

A possible explanation is that the core effects for the form factors were partially canceled out in the *f* = 12 molecules due to other intermolecular effects only present in high-armed stars. A form factor maximum smaller than expected implies a correction leading to a faster approach to the MTS in the common representation which is in qualitative agreement with the observed trend. Therefore, we would need to substitute *f_p_* with a greater factor, *f_hb_*, if we want to apply the adequate correction in the RPA description to align the data of our highly armed stars with the results obtained for the other systems. 

**Figure 4:**
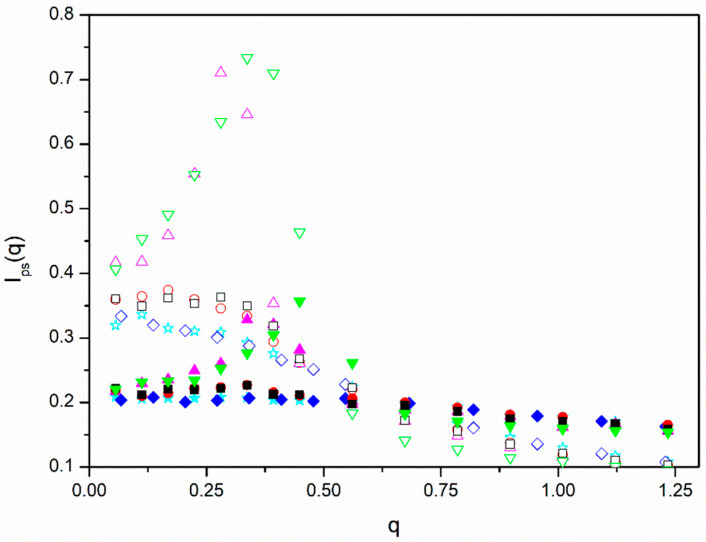
Scattering functions obtained with identical optical contrast factors for A and B units with respect to the solvent, Equation (9), see text, vs. the scattering variable, *q*, for systems without segregation, *ε* = 0 case. Blue rhombuses, *N =* 144, *f = 2*; red circles: *N =* 145, *f =* 6; green triangles down: *N =* 73, *f =* 12; cyan stars: *N =* 72, *f = 2*; black squares, *N =* 73, *f =* 6; magenta triangles up: *N =* 145, *f =* 12. Filled symbols correspond to *Φ* ≅ 0.275 and open symbols correspond to *Φ* ≅ 0.135.

We investigated if there are spatial correlations, between positions of different molecules as it can be expected from simulations for non-diluted *f* = 12 homopolymer star molecule solutions obtained some time ago with the BFM [23] and, also, from theoretical predictions related to the discontinuity of the osmotic pressure at the overlapping concentration of non-dilute solutions [31]. They manifest themselves in terms of a peak in the scattering structure factor obtained assuming the same scattering for all units with respect to the solvent, *I_ps_*(*q*), according to Equation (9). In Figure 4, we plotted our results for *I_ps_*(*q*) obtained for the different molecules with *ε* = 0.

We observe that the scattering functions are practically flat for the linear and *f* = 6 stars and the highest density, showing that these systems are similar to melts. The data for the same molecules and the smaller density exhibit a flat peak for *q* ≅ 0 with a decrease when higher values of *q* are considered. This behavior represents the intermediate behavior between the scattering shown by dilute molecules and melts that can be characterized by the size of the blobs that describe semidilute solutions [12]. It can be observed that the decrease is slower in the case of the *f* = 6 with a melt-like density of beads in the cores and smaller blobs. The *f* = 12 molecules, however, show sharp peaks at intermediate values of *q*, with a dramatic increase at small values corresponding to the range where the maximum in the *I_AB_*(*q*) curves are located. Therefore, we conclude that our *f* = 12 star systems show a crystal-like structure. It can also be observed that the intensity of the peaks has a noticeable dependence on density, being flatter for the higher density that is closer to the melt state.

**Figure 5:**
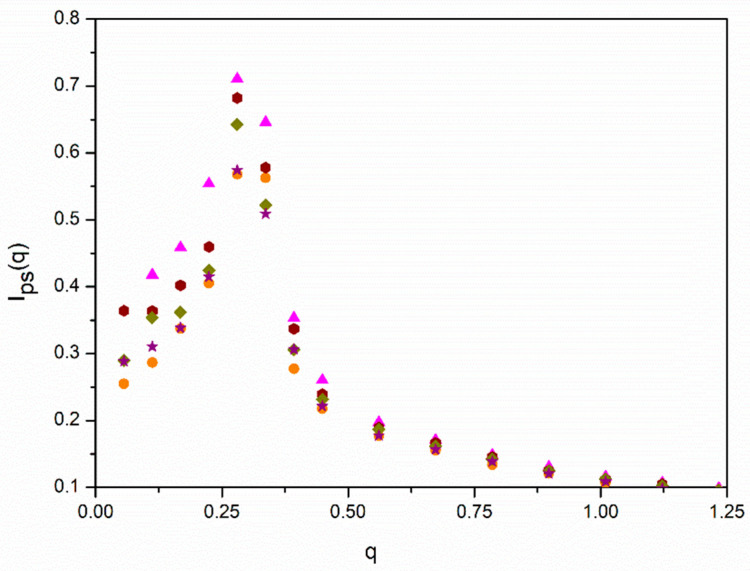
Scattering functions obtained with identical optical contrast factors for A and B units with respect to the solvent, Equation (9), see text, vs. the scattering variable, *q*, for the *N* = 145, *f* = 12, *Φ* ≅ 0.135 case and different values of the segregation parameter. Magenta triangles up, *ε* = 0; wine hexagons: *ε =* 0.15; dark yellow rhombuses: *ε* = 0.10; purple stars: *ε* = 0.15; orange circles: *ε* = 0.20.

In Figure 5, we plotted the data for *I_ps_*(*q*) obtained with different values of *ε* for one of the systems with the lower density where the peak for *ε* = 0 is particularly prominent. We can distinguish a slight decrease in the peak for higher values of *ε*, showing that segregation tends to moderately relax the spatial ordering of the molecules.

However, it is possible that spatial ordering may reinforce segregation for increasing values of *ε*. Intuitively, one can expect that the formation of mesophase structures is easier for ordered systems. We explored the possibility that the relative increase in *I_AB_*(*q_max_*,*ε*) for the *f* = 12 systems can be described assuming that the hypothetical factor *f_hb_* described above can be related with our results obtained with *ε* = 0 for *I_ps_*(*q*). The total intensities for *q* = 0 and *q_max_* obtained for the *f* = 12 cases are included in Table 3. The relative increase in the intensity at *q_max_* has a noticeable dependence on density, confirming that the smaller density corresponds to a semidilute solution that is closer to the overlapping concentration. We included in Figure 2 the results corresponding to miktoarms with the highest number of arms, *f* = 12, but using the alternative plots of *I_AB_*(*q_max_*,*ε* = 0)/*I_AB_*(*q_max_*,*ε*) vs. *f_hb_εNΦ*, with *f_hb_ = f_p_I_ps_*(*q_max_*)/*I_ps_*(*q* = 0). This factor tries to include a correction in the form factor maximum due to the non-uniform distribution of the star positions. It is observed that we obtain a reasonable alignment of the *f* = 12 data with the rest with the help of this purely tentative type of description. It should be noted that factor *f_hb_* is concentration-dependent.

## 4. Summary and Conclusions

The present simulations study segregation between blocks for several non-diluted solutions of diblock copolymers and miktoarms, or Janus star polymers, with *f* = 6, 12 number of arms in a solvent of good quality for the two types of blocks. Although different properties are calculated, particular attention is paid to characterize the approach to the MST through the scattering structure function obtained by setting opposite optical contrast factors for the different units A or B. It is verified that the dependence of the minimum value of the inverse scattering function with the segregation variable employed in the simulations (proportional to the FH parameter) follows a reasonably good universal decreasing linear function for low values of *ε* and the simulation data corresponding to different systems can be grouped into a single plot. An extrapolation of the *I_AB_*(*q_max_*)^−1^ data to zero determines the value of *ε* that corresponds to the MST in the melt state for the BFM model. However, the *f* = 12 miktoarm results for *I_AB_*(*q_max_*)^−1^ show a systematically faster approach to the MTS, and they can be included in the universal representation only with the help of an additional tentative modification related to the spatial correlation between star molecules.

## Figures and Tables

**Table 1:** Values of conformational properties related to the degree of segregation, type of molecule, density and number of units.

	*ε*	*a_r_*	10^2^*C_AB_*	*A*	$R_{g}^{2}$	$\langle R_{ee}^{2}\rangle$	10^−4^<*E_rep_*>/*ε*
							
*Φ* ≅ 0.275							
*f =* 2, *N =* 144							
	0	0.225	−2.0	0.528	285	1727	1.4
	0.01	0.224	−2.6	0.528	283	1706	2.5
	0.015	0.223	−2.9	0.528	282	1700	4.7
	0.03	0.221	−6.0	0.536	282	1700	2.1
	0.05	0.218	−10	0.543	290	1762	1.6
	0.07	0.215	−15	0.562	305	1864	1.06
	0.10	0.211	−21	0.582	324	2015	0.83
	0.15	0.201	−24	0.591	330	2060	0.460
	0.20	0.192	−26	0.599	331	2087	0.350
							
*f =* 6, *N =* 145	0	0.231	−1.0	0.156	142	295	3.2
	0.03	0.212	−1.2	0.156	141	292	2.4
	0.05	0.201	−1.5	0.155	141	292	2.2
	0.07	0.191	−2.0	0.155	142	291	2.0
	0.10	0.176	−3.1	0.157	144	293	1.4
	0.15	0.153	−5.1	0.167	150	304	0.89
	0.20	0.133	−6.3	0.176	156	315	0.61
							
*f =* 12, *N =* 145	0	0.236	−0.78	0.061	87	162	4.3
	0.03	0.220	−1.1	0.061	88	162	3.1
	0.05	0.210	−1.34	0.061	88	162	2.8
	0.075	0.197	−1.8	0.060	88	162	2.4
	0.10	0.184	−2.2	0.061	89	163	2.2
	0.15	0.161	−3.5	0.062	90	164	1.54
	0.20	0.140	−4.7	0.065	91	166	1.10
	0.25	0.122	−5.6	0.069	93	169	0.88
	0.30	0.106	−6.2	0.072	94	173	0.63
	0.40	0.079	−6.8	0.078	96	176	0.48
							
*f =* 2, *N =* 72	0	0.227	−2.04	0.526	137	825	1.78
*f =* 6, *N =* 73	0	0.234	−1.12	0.147	71	143	3.3
*f =* 12, *N =* 73	0	0.247	−1.02	0.052	44.9	79.6	4.2
							
*Φ* ≅ 0.135							
*f =* 2, *N =* 144	0	0.249	−4.46	0.535	345	2121	0.45
*f =* 6, *N =* 145	0	0.253	−1.072	0.148	166	345	0.57
*f =* 12, *N =* 145	0	0.259	−0.99	0.054	102	191	0.82
							
*f =* 2, *N =* 72	0	0.262	−3.32	0.532	160	973	0.32
*f =* 6, *N =* 72	0	0.263	−1.47	0.138	81	164.1	0.69
*f =* 12, *N =* 72	0	0.271	−1.18	0.047	48.5	89	1.2

**Table 2:** Values of the form factor according to the RPA and simulation results obtained from the two different models explained in the text for the systems with the highest density, *Φ* ≅ 0.275.

	*Ideal Limit*	*P_AB_*(*q*), Equation (10)	<*P_AB_*(*q_max_*)>
f = 2	0.190		
*N =* 144		0.205	0.204
*N =* 72		0.208	0.198
f = 6	0.063		
*N =* 145		0.086	0.078
*N =* 73		0.091	0.086
f = 12	0.032		
*N =* 145		0.048	0.048
*N =* 73		0.05	0.05

**Table 3:** Scattering functions at *q* = 0 and *q_max_* obtained assuming the same optical contrast factors for A and B units with respect to the solvent corresponding to the *f =* 12-star molecules without segregation and with different densities and number of units.

*f* = 12	*I_ps_*(*q*_max_)	*I_ps_*(*q* = 0)
*Φ* ≅ 0.275, *N =* 145	0.30	0.23
*Φ* ≅ 0.275, *N =* 73	0.30	0.22
*Φ* ≅ 0.135, *N =* 145	0.71	0.42
*Φ* ≅ 0.135, *N =* 73	0.73	0.41

## Data Availability

Not applicable.
